# Effect of C-terminus Conjugation via Different Conjugation Chemistries on In Vivo Activity of Albumin-Conjugated Recombinant GLP-1

**DOI:** 10.3390/pharmaceutics13020263

**Published:** 2021-02-15

**Authors:** Junyong Park, Mijeong Bak, Kiyoon Min, Hyun-Woo Kim, Jeong-Haeng Cho, Giyoong Tae, Inchan Kwon

**Affiliations:** 1 Department of Biomedical Science and Engineering, Gwangju Institute of Science and Technology (GIST), Gwangju 61005, Korea; happydragon@gist.ac.kr; 2 Gwangju Institute of Science and Technology (GIST), School of Materials Science and Engineering, Gwangju 61005, Korea; al4527@gm.gist.ac.kr (M.B.); kymin324@gist.ac.kr (K.M.); gytae@gist.ac.kr (G.T.); 3 R&D Center, ProAbTech Co., Ltd., Gwangju 61005, Korea; kimhw@proabtech.com (H.-W.K.); nucleic@proabtech.com (J.-H.C.)

**Keywords:** plasma half-life extension, albumin conjugation, in vivo glucose-lowering activity, glucagon-like peptide-1

## Abstract

Glucagon-like peptide-1 (GLP-1) is a peptide hormone with tremendous therapeutic potential for treating type 2 diabetes mellitus. However, the short half-life of its native form is a significant drawback. We previously prolonged the plasma half-life of GLP-1 via site-specific conjugation of human serum albumin (HSA) at position 16 of recombinant GLP-1 using site-specific incorporation of p-azido-phenylalanine (AzF) and strain-promoted azide-alkyne cycloaddition (SPAAC). However, the resulting conjugate GLP1_8G16AzF-HSA showed only moderate in vivo glucose-lowering activity, probably due to perturbed interactions with GLP-1 receptor (GLP-1R) caused by the albumin-linker. To identify albumin-conjugated GLP-1 variants with enhanced in vivo glucose-lowering activity, we investigated the conjugation of HSA to a C-terminal region of GLP-1 to reduce steric hindrance by the albumin-linker using two different conjugation chemistries. GLP-1 variants GLP1_8G37AzF-HSA and GLP1_8G37C-HSA were prepared using SPAAC and Michael addition, respectively. GLP1_8G37C-HSA exhibited a higher glucose-lowering activity in vivo than GLP1_8G16AzF-HSA, while GLP1_8G37AzF-HSA did not. Another GLP-1 variant, GLP1_8A37C-HSA, had a glycine to alanine mutation at position 8 and albumin at its C-terminus and exhibited in vivo glucose-lowering activity comparable to that of GLP1_8G37C-HSA, despite a moderately shorter plasma half-life. These results showed that site-specific HSA conjugation to the C-terminus of GLP-1 via Michael addition could be used to generate GLP-1 variants with enhanced glucose-lowering activity and prolonged plasma half-life in vivo.

## 1. Introduction

Diabetes mellitus is one of the most common chronic diseases worldwide, involving the loss of control of blood glucose levels, which results in a continuously elevated glucose concentration. The global prevalence of diabetes mellitus among adults over 18 years of age increased from 4.7% in 1980 to 8.5% in 2014 [1]. In 2016, an estimated 1.6 million deaths were directly caused by diabetes [2]. To prevent these complications, controlling the blood glucose levels of patients with diabetes is of great importance. Glucagon-like peptide-1 (GLP-1) is an essential hormone that contributes to the regulation of blood glucose levels. After secretion from L-cells of the intestine [3], GLP-1 is directed toward many organs to reduce the blood glucose levels, including the pancreas, heart, muscles, kidneys, liver, and even the brain [4]. The activity of GLP-1 is mediated by its binding to the GLP-1 receptor (GLP-1R) on the membrane of cells found in various organs, including pancreatic beta cells [5,6,7]. After GLP-1R is stimulated by GLP-1 binding, it activates the G-protein and upregulates cyclic adenosine monophosphate (cAMP) inside the cell and causes other synergistic effects, which leads to proliferation of pancreatic beta cells, enhanced insulin production inside the pancreatic beta cells, and insulin secretion to control the blood glucose levels [8,9,10,11]. 

Because of its rapid renal clearance due to its small size (~3 kDa) and its vulnerability to proteolytic cleavage by dipeptidyl peptidase-IV (DPP-IV), GLP-1 is reported to have an extremely short half-life in the body (~2–3 min). Although GLP-1 exerts significant effects in an individual’s body, its short half-life is a significant drawback. Many studies have been conducted in effort to overcome the hurdles of GLP-1 and to develop it as a medication. Traditional efforts to increase the size of small therapeutic proteins have included polyethylene glycol (PEG) conjugation. However, PEG conjugation has been reported to cause several complications, including renal accumulation and potential immunogenicity [12,13,14,15,16,17]. Recently, serum albumin has been proposed as a good half-life extender, as it is a naturally abundant protein that is amenable to chemical conjugation via the free thiol on cysteine 34. Furthermore, albumin can bind to the neonatal Fc receptor (FcRn) in an acidic environment, which protects it from intracellular degradation and allows it to be recycled into the extracellular space. Accordingly, we and other researchers have attempted to use direct chemical conjugation or indirect binding of proteins to albumin via albumin-binding ligands to prolong the plasma half-life of the proteins [18,19]. To address the short half-life of GLP-1 resulting from its cleavage by DPP-IV, the GLP-1 backbone of GLP-1R agonists has been changed. For instance, the alanine at position 8 of GLP-1 is cleaved by DPP-IV [20] and the cleaved GLP-1 fragment (residues 9‒39) is known for its low biological activity as the N-terminal moiety of GLP-1 is important for receptor activation [21]. Therefore, substitution of the alanine with glycine, or any other amino acid including non-natural amino acids (NNAAs), has been used in attempt to confer resistance to DPP-IV-induced cleavage [22,23].

**Figure 1:**
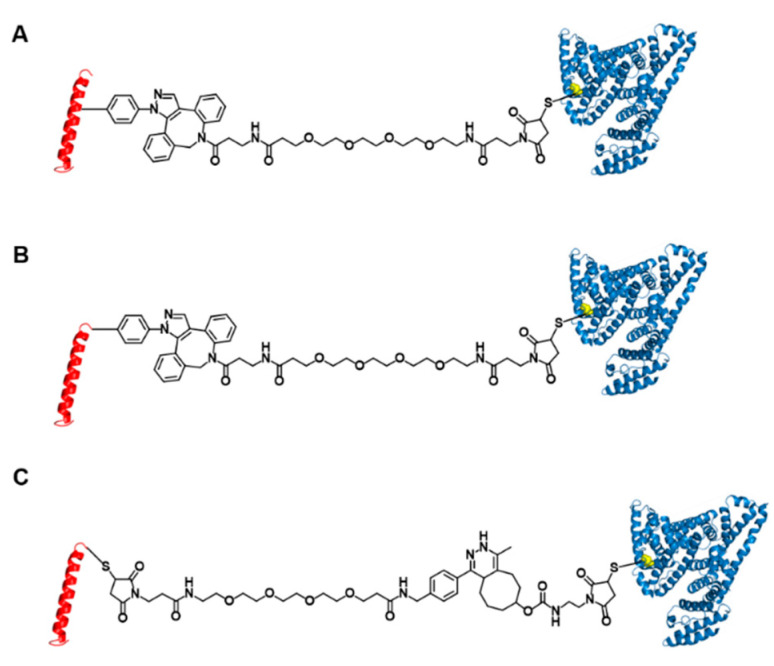
Structures of three glucagon-like peptide-1 (GLP-1)-human serum albumin (HSA) variants. GLP-1 variants conjugated to HSA at position 16 (**A**) and position 37 (**B**) using strain-promoted azide-alkyne cycloaddition and Michael addition. (**C**) GLP-1 variants conjugated to HSA at position 37 using inverse-electron demand Diels-Alder reaction and Michael addition.

We previously reported the site-specific HSA conjugation to GLP-1 by expressing recombinant GLP-1 variants bearing an NNAA as an albumin conjugation site fused to superfolder green fluorescent protein (sfGFP) in genetically engineered *Escherichia coli* [24]. Two of the resulting GLP-1 variants with human serum albumin (HSA) conjugated at positions 16 and 28, respectively, exhibited significantly prolonged plasma half-lives in mice (~8 h) [24]. Although we carefully selected the albumin conjugation site on GLP-1 in order for it not to interfere with the interactions of position 16 of GLP-1with GLP-1R (Figure 1A), in vitro and in vivo glucose-lowering activities of the resulting albumin conjugates were only moderate [24]. Enhancing the biological potency of a drug can help reduce the amount of injected drug needed for efficacy, thereby contributing to a reduction in production cost. 

**Figure 2:**
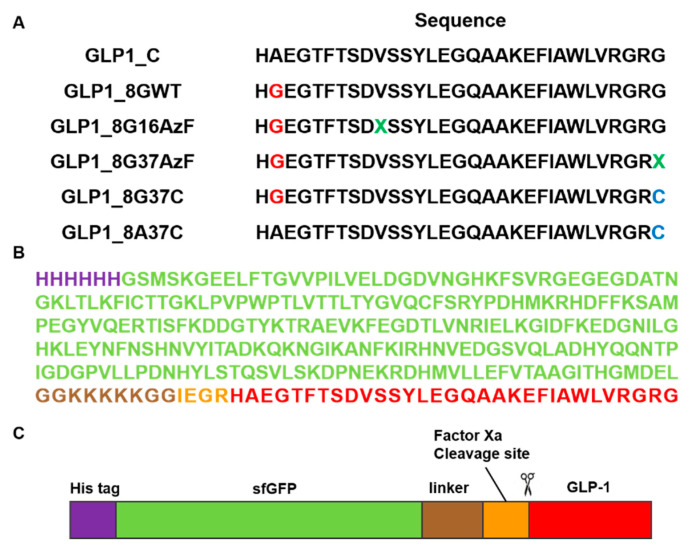
Construction of the GLP-1 and superfolder green fluorescent protein (sfGFP)-fused GLP-1 (sfGFP-GLP1) variants. (**A**) The amino acid sequences of six GLP-1 variants. The green-colored X notated at GLP1_8G16AzF and GLP1_8G37AzF was the incorporation site of the non-natural amino acid, AzF. (**B**) The amino acid sequence and (**C**) protein features of sfGFP-GLP1_C with the polyhistidine-tag (purple), sfGFP (green), linker (brown), factor Xa cleavage site (orange), and GLP1_C (red).

In the current study, we investigated the effects of albumin conjugation sites and conjugation chemistry on in vivo blood-lowering activities with the ultimate goal of designing albumin-GLP1 conjugates with enhanced therapeutic potency. Based on mutation studies, an albumin conjugate site at GLP1 position 16 was selected in a previous study to minimize the perturbation of interactions between the GLP-1 variant and GLP-1R [24]. However, considering the bulky structure of albumin, we hypothesize that the albumin conjugated to GLP-1 may to some extent block the binding of the conjugate to GLP-1R. The crystal structure of GLP-1 complexed with GLP-1R (PDB ID: 5VAI) indicates that albumin conjugated to the C-terminal region of GLP-1 causes less steric hindrance with GLP1-1R than that of albumin conjugated to the middle region of GLP-1. Therefore, we prepared GLP1_8G37AzF-HSA, a new GLP1-HSA conjugate generated by HSA conjugation to the C-terminal region of GLP-1 (Figure 1B), as well as GLP1_8G16AzF-HSA, a GLP1-HSA conjugate generated by HSA conjugation to position 16 of GLP-1 [24]. In addition to the albumin conjugate site, we hypothesized that the conjugation chemistry may also affect in vivo activity of GLP1-HSA conjugates. In a previous study, we used strain-promoted azide-alkyne cycloaddition (SPAAC) to couple azido groups to dibenzocyclooctyne (DBCO), which resulted in a very bulky four-ring structure. Such a bulky structure could reduce the binding affinity of the conjugate to GLP-1R by either direct or indirect interactions with GLP-1R. Therefore, we also prepared the GLP1-HSA conjugate GLP1_8G37C-HSA by conjugating HSA to a GLP-1 variant bearing a cysteine at position 34 (GLP1_8G37C) via a Michael addition reaction (Figure 1C). The cysteine at position 34 was often used for albumin modification, because it is away from FcRn binding site [24]. The effects of alanine and glycine at position 8 of GLP-1 were also investigated in the current study. Replacing the alanine at position 8 with glycine prevented DPP-IV-mediated cleavage of GLP-1. However, there are reports that this mutation results in a mild loss of biological activity in vitro (4‒10-fold decrease) [23,25,26]. To further investigate this, we substituted glycine with alanine at position 8 on GLP1_8G37C, resulting in GLP1_8A37C (Figure 2A). All GLP-1 variants were expressed in *E. coli* using sfGFP as a fusion tag (Figure 2B,C), as previously reported [24]. After expression of each GLP-1 protein, albumin conjugation was completed, followed by proteolytic cleavage using factor Xa to dissociate the fusion tag from the desired conjugate (Figure 2B,C). We subsequently investigated the in vivo activities, in vivo half-life, and in vitro activities of each variant. 

## 2. Materials and Methods

### 2.1. Materials

Polypropylene columns and nickel-nitrilotriacetic acid (Ni-NTA) agarose beads were purchased from Qiagen (Valencia, CA, USA). Trans-cyclooct-2-ene maleimide (TCO-MAL) and methyltetrazine-PEG4-maleimide (TET-PEG4-MAL) were obtained from Futurechem (Seoul, Korea). Dibenzocyclooctyne-PEG4-maleimide (DBCO-PEG4-MAL) was purchased from Click Chemistry Tools LLC (Scottsdale, AZ, USA). p-Azido-l-phenylalanine (AzF) was purchased from Chem-Impex International (Wood Dale, IL, USA). Disposable PD-10 desalting columns with Sephadex G-25 resin, Ion exchange chromatography columns (HiTrap Q HP and HiTrap SP HP) were obtained from GE Healthcare (Little Chalfont, Buckinghamshire, UK). Factor Xa was obtained from New England Biolabs (Ipswich, MA, USA). The GLP-1_WT and GLP-1_8GWT peptides were synthesized by GenScript (Piscataway, NJ, USA). Vivaspin 6 concentrator and 10,000 MWCO were purchased from Sartorius (Weender Landstraße, Göttingen, Germany). The mouse anti-GLP-1 monoclonal antibody was purchased from Thermo Fisher Scientific (Waltham, MA, USA). Rabbit anti-albumin polyclonal antibody was purchased from Sigma-Aldrich (St. Louis, MO, USA). The anti-mouse IgG, horseradish peroxidase (HRP)-linked antibody was purchased from Cell Signaling Technology (Beverly, MA, USA). Human embryonic kidney 293 (HEK293) cells were obtained from American Type Culture Collection (ATCC; Manassas, VA, USA). Fetal bovine serum and antibiotic-antimycotic for cell culture were purchased from Gibco (Gaithersburg, MD, USA). Iscove’s modified Dulbecco’ s medium and the Transfection Reagent Kit for the in vitro assays were purchased from Sigma Aldrich. DNA transfection reagent (X-tremeGENE HP) was obtained from Roche Diagnostics GmbH (Mannheim, Germany). The cAMP assay kit was obtained from R&D Systems (Minneapolis, MN, USA). Unless otherwise noted, all other chemicals were obtained from Sigma-Aldrich.

### 2.2. Preparation of sfGFP and GLP-1 Fusion Protein

Construction of the pQE80-sfGFP-GLP1_C plasmid was described in a previous report [24]. This plasmid encodes a fusion protein of sfGFP and GLP1_C and was used as a template for mutagenesis. To confer resistance to proteolytic degradation by DPP-IV, a PCR-based site-directed mutagenesis was used to replace the alanine at position 8 of GLP-1 with glycine by using primers A8G_F and A8G_R. For site-specific incorporation of AzF, the V16 and G37 sites of GLP-1 were mutated to an amber codon (TAG). The final plasmids generated were pQE80-sfGFP-GLP1_8G16Amb and pQE80-sfGFP-GLP1_8G37Amb, which were obtained by a PCR-based site-directed mutagenesis using primers V16AzF_F, V16AzF_R, G37AzF_F, and G37AzF_R (Table S1). To generate the 37C variants pQE80-sfGFP-GLP1_8A37C and pQE80-sfGFP-GLP1_8G37C, cysteine was substituted for glycine at position 37 of GLP-1 using primers G37C_F and G37C_R (Table S1). The pEvol-pAzFRS.1.t1 plasmid encoding the orthogonal pair MjTyrRS/MjtRNA_CUA_ was obtained from Addgene (Addgene plasmid #73547, Watertown, MA, USA) [27]. To express the AzF-incorporated fusion protein, one of the amber codons-containing plasmids and pEvol-pAzFRS.1.t1 were co-transformed into C321ΔA.exp. *E. coli* cells (Addgene plasmid #49018) [28]. For the expression of variants 8A37C and 8G37C, each plasmid (pQE80-sfGFP-GLP1_8A37Cys and pQE80-sfGFP-GLP1_8G37Cys) was transformed into TOP10 *E. coli* cells.

The overnight cultures of C321ΔA.exp cells containing one of the amber codons-containing plasmids and pEvol-pAzFRS.1.t1 were inoculated into 200 mL of 2ⅹYT media containing ampicillin (100 μg/mL) and chloramphenicol (35 μg/mL) and incubated at 37 °C with 210 rpm shaking. When the absorbance at 600 nm (OD_600_) reached 0.4, AzF was supplemented to the culture to a final 1 mM concentration. When the OD_600_ reached 0.5, sfGFP-GLP1 fusion protein expression was induced by the addition of isopropyl-β-D-thiogalactopyranoside (IPTG) and l-(+)-arabinose to the culture at final 1 mM and 0.2% (*w/v*) concentrations, respectively. The cells were cultured at 25 °C with 210 rpm shaking and collected after 15 h by centrifugation (6000 rpm, 15 min). The expression of sfGFP-GLP1_8G37C and sfGFP-GLP1_8A37C were performed as described above, except TOP10 *E. coli* was used for pQE80-sfGFP-GLP1_8G37Cys and TOP10 *E. coli* was used for pQE80-sfGFP-GLP1_8A37Cys, and chloramphenicol, AzF, and l-(+)-arabinose were not added.

The sfGFP-GLP1 fusion protein variants containing AzF were purified using Ni-NTA resin (QIAGEN, Hilden, Germany) and an N-terminal hexahistidine-tag according to the manufacturer’s manual. The purified sfGFP-GLP1 was then exchanged with 20 mM Tris (pH 8.0), and then subjected to anion exchange chromatography using a HiTrap Q HP column. The sfGFP-GLP1_8G37C and sfGFP-GLP1_8A37C were purified by similar affinity purification steps except 5 mM tris(2-carboxyethyl)phosphine (TCEP) was added to all buffers. Subsequently, sfGFP-GLP1_8A37C and sfGFP-GLP1_8G37C were functionalized by the thiol-maleimide reaction using cysteine at position 37 of GLP-1. The purified proteins were buffer-exchanged with PBS (pH 7.3) and mixed with TET-PEG4-MAL (1:4 molar ratio). Unreacted linker was removed by desalting with 20 mM Tris (pH 8.0) using a PD-10 column after 3 h. The functionalization process ended with the production sfGFP-GLP1_8A37C-TET and sfGFP-GLP1_8G37C-TET, respectively.

### 2.3. Preparation of GLP1_8G16AzF-HSA, GLP1_8G37AzF-HSA, GLP1_8G37C-HSA, and GLP1_8A37C-HSA Conjugates

HSA-DBCO was prepared as described previously [24]. The prepared HSA-DBCO was conjugated with sfGFP-GLP1_8G16AzF and sfGFP-GLP1_8G37AzF and HSA-TCO was conjugated at room temperature (RT) overnight with sfGFP-GLP1_8G37C-TET and sfGFP-GLP1_8A37C-TET (1:2 molar ratio). Each mixture was buffer exchanged into 20 mM sodium phosphate buffer (pH 6.0). Then, cation exchange chromatography was performed to remove the unreacted HSA and sfGFP-GLP1 by using a HiTrap SP HP column with 20 mM sodium phosphate (pH 6.0).

The separated sfGFP-GLP1-HSA was buffer-exchanged into a buffer (2 mM CaCl_2_, 10 mM NaCl, 20 mM Tris; pH 8) and then concentrated (final 10 μM concentration) using a Vivaspin 6 concentrator, 10,000 MWCO. The sfGFP-GLP1-HSA was incubated with 1/200 (*w/w*) Factor Xa protease for 18 h at room temperature. The Factor Xa reaction was terminated by the addition of dansyl-Glu-Gly Arg-chloromethyl ketone (0.1 mg/mL). The Factor Xa-processed conjugate solution was further purified to obtain the GLP1-HSA by the buffer-exchange into 20 mM Bis-Tris (pH 6.0) and anion exchange chromatography using a HiTrap Q HP column.

### 2.4. Labeling of Linker Conjugated sfGFP-GLP1_8G37C by Inverse Electron-Demand Diels-Alder Reaction (IEDDA)

For labeling, 20 μM sfGFP-GLP1_8G37C or 20 μM sfGFP-GLP1_8G37C-TET in PBS (pH 7.4) was mixed with TCO-cy5.5 dye at a molar ratio of 1:5 for 2 h. As a control, sfGFP-GLP1_8G37C-TET without dye was prepared. Each mixture was subjected to sodium dodecyl sulfate-polyacrylamide gel electrophoresis (SDS-PAGE) and fluorescence imaging using a Bio-Rad ChemiDoc XRS + Imaging System (Bio-Rad, Hercules, CA, USA).

### 2.5. Mass Spectrometric Analysis

All analytes were prepared using a ZipTip C18 system in accordance with the manufacturer’s manual. After ZipTip processing, GLP1_8G37AzF, GLP1_8G37C, and GLP1_8A37C were mixed 1:1 (*v:v*) with α-cyano-4-hydroxy cinnamic acid (HCCA)-saturated TA30, which was a solution (30% acetonitrile; 0.1% trifluoroacetic acid). The GLP1_8G37AzF-HSA, GLP1_8G37C-HSA, and GLP1_8A37C-HSA after ZipTip processing were mixed with 20 mg/mL 2,5-dihydroxybenzoic acid (DHB) in TA30. Each mixture placed on a polished steel plate and mass characterization was conducted using Autoflex matrix-assisted laser desorption-ionization/time-of-flight mass spectroscopy (MALDI-TOF MS) and the corresponding flexControl software (Bruker Daltonics, Bremen, Germany).

### 2.6. Enzyme-Linked Immunosorbent Assay (ELISA) of GLP1-HSA Conjugate

The immunoplate was coated with 5 μg/mL anti-albumin rabbit antibody in 0.1 M bicarbonate pH 9.6 at 4 °C overnight and then blocked with 5% skim milk. After removal of the solution from each well, the serum samples were diluted in 5% skim milk and incubated in each well for 2 h at room temperature. The GLP1-HSA variant was used as a calibration standard. After washing each well, 1 μg/mL anti-GLP-1 mouse antibody diluted in 5% skim milk was incubated at RT for 2 h. Each well of the plate was washed and then incubated with an anti-mouse HRP-conjugated IgG antibody diluted 1/3000 in 5% skim milk at RT for 1 h. Once again after washing, 3, 3′,5, 5-tetramethylbenzidine was applied and incubated at room temperature for a short time. The reaction was quenched using 100 μL of 2 M H_2_SO_4_. Then, the absorbance at 450 nm was monitored using a Synergy™ microplate reader (BioTek, Winooski, VT, USA).

### 2.7. Pharmacokinetic Studies of GLP1-HSA Conjugates

In vivo pharmacokinetic studies were conducted using eight-week-old female BALB/c mice (DBL, Korea). The mice were maintained in a 12-h light/12-h dark cycle and freely accessed to water and food. All animal protocols were approved by the Animal Ethics Committee of GIST (Approval number: GIST-2019-071 (4 October 2019)) in accordance with the Guidelines for Care and Use of Laboratory Animals proposed by the Gwangju Institute of Science and Technology (GIST). The mice were randomly divided into two groups (n = 4/group). Either GLP1_8G37C-HSA or GLP1_8A37C-HSA (10 nmol/kg dose) was intravenously administered to the respective groups of mice. Blood samples (<70 μL) were collected from the retroorbital venous sinus at 0.16, 1, 3, 6, 12, and 24 h after conjugate administration. The acquired blood samples were placed at RT for 30 min and then centrifuged at 4 °C (2500 rpm, for 10 min). The serum was collected, and the samples stored at −20 °C until analyzed. Plasma concentrations of the GLP1-HSA conjugate at each time point was measured in triplicate by ELISA.

### 2.8. In Vivo Intraperitoneal Glucose Tolerance Test (IPGTT)

Normal seven-week-old C57BL/6J male mice (DBL, Korea) were randomly divided into six groups (*n* = 6/group). The mice were fasted for 3–6 h prior to the experiment. GLP1_C (30 nmol/kg), the GLP1-HSA variants (30 nmol/kg), or saline were subcutaneously administered 20 min before an intraperitoneal injection of glucose (1.5 g/kg). Blood samples were collected from the tail and glucose levels were determined using an Accu-Check Guide (Roche Diabetes Care, Indianapolis, IN, USA). 

### 2.9. In Vitro Activity Assay

An in vitro activity assay for GLP1_C and its variants was performed as described in our previous study [24]. Briefly, 10,000 HEK293 cells were seeded per well onto a 48-well plate and incubated at 37 °C under 5% CO2 for 16 h. The plasmid pcDNA3.1-GLP-1R_tango (Addgene, Cambridge, MA, USA) [29] and transfection reagent were then mixed in serum-free medium for 20 min, in accordance with the manufacturer’s recommendation. Then, 30 μL of the mixture was dropped into each well and the plate was incubated for 48 h at 37 °C. GLP1_C or variant peptides were diluted 10-fold with media and added to the transfected cells for 15 min. The levels of cAMP in the cell lysate were measured using the cAMP Parameter Assay Kit following the manufacturer’s protocol. The synthetic GLP1_C peptide solutions at required concentrations were prepared using deionized water just prior to performing the assay. The values were normalized to the amount of cAMP secreted with GLP1_C of 10^–6^ M and converted to % activity. The half maximal effective concentrations (EC_50_) of each curve were estimated from a dose–response curve using OriginPro software. Absorbance at 450 nm was monitored using a Varioskan Lux microplate reader (Thermo Fisher Scientific).

## 3. Results and Discussion

### 3.1. Preparation of C-terminus-Modified GLP-1 Varints

**Figure 3:**
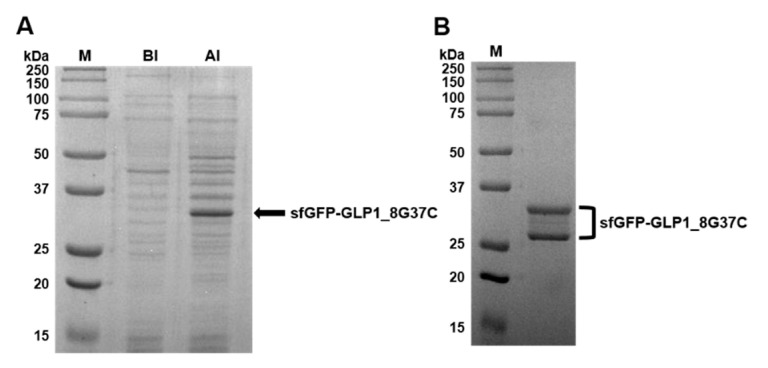
Expression, purification, and confirmation of C-terminal cysteine-substituted sfGFP-GLP-1_8G37C. (**A**) SDS-PAGE protein gel images of cell lysates before induction (BI) and after induction (AI). A prominent band expected to be sfGFP-GLP1_8G37C is observed (arrow). (**B**) Purified sfGFP-GLP1_8G37C in an SDS-PAGE gel after Ni-NTA affinity chromatography.

Preparation of sfGFP-GLP1_8G37C was representatively described in detail. Comparison of cell lysates before induction (BI) and after induction (AI) of sfGFP-GLP1_8G37C showed a prominent new band in the A.I. sample in a Coomassie blue-stained gel (Figure 3A). The protein band was shown between 25 kDa and 37 kDa molecular markers, consistent with the expected molecular weight of sfGFP-GLP-1_8G37C (~32 kDa). Purified sfGFP-GLP1_8G37C was obtained by Ni-NTA affinity chromatography (Figure 3B), and its size shown to be between 25 kDa and 37 kDa. However, two bands were observed for the purified sfGFP-GLP1_8G37C, which we speculated was a result of different oxidation status of sfGFP-GLP1_8G37C [30]. The preparation of sfGFP-GLP1_8A37C was performed in a similar manner to that of sfGFP-GLP1_8G37C and the preparation of sfGFP-GLP1_8G16AzF was performed as previously reported [24]. 

To append the sfGFP-GLP1_8G37C functional group for reaction with HSA, TET-PEG4-MAL was reacted with sfGFP-GLP1_8G37C to generate sfGFP-GLP1_8G37C-TET. We chose this linker in order to use the IEDDA, which is known for its high reactivity compared to that of the formerly used bioorthogonal SPAAC [31]. The reactivity of methyltetrazine was evaluated by conjugating the linker to a fluorescent dye. Briefly, the sfGFP-GLP1_8G37C-TET conjugate was reacted with TCO-Cy5.5 dye and the in-gel fluorescence of the reaction mixture analyzed (Figure S1). In the Coomassie-stained gel, the upper band showed a mild upward shift after conjugation with TET-PEG4-MAL. In contrast, migration of the lower band, which we speculated to be the oxidized form of sfGFP-GLP-1_8G37C, did not change after conjugation with the TET-PEG4-MAL linker. This was attributed to the absence of a reduced cysteine, which was required to undergo the reaction with maleimide. TCO-Cy5.5 dye-labeled sfGFP-GLP-1_8G37C-TET showed prominent fluorescence, whereas sfGFP-GLP-1_8G37C, which could not react with the TCO-Cy5.5, failed to show fluorescence after incubation with the dye. Labeling the conjugate with the dye confirmed the sfGFP-GLP1_8G37C-TET construct. 

**Figure 4:**
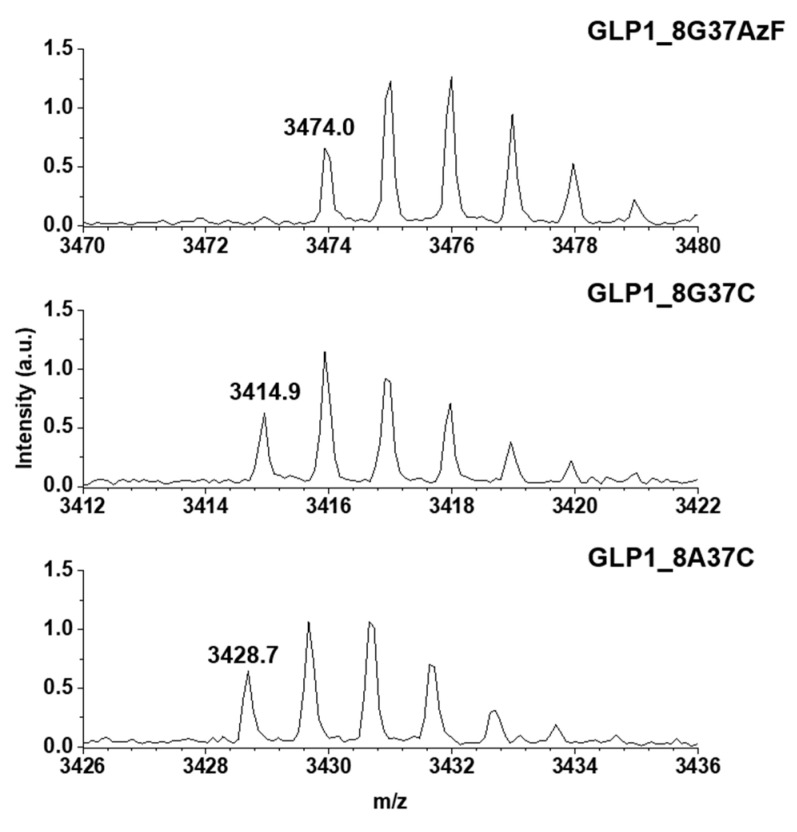
Monoisotopic mass confirmation of GLP1_8G37AzF, GLP1_8G37C, and GLP1_8A37C by matrix-assisted laser desorption/ionization time-of-light mass spectroscopy (MALDI-TOF MS). The monoisotopic mass to charge ratio for GLP1_8G37AzF, GLP1_8G37C, and GLP1_8A37C were 3474.0, 3414.9, and 3428.7 m/z, respectively.

Meanwhile, the construction of GLP1_8G37AzF, GLP1_8G37C, and GLP1_8A37C were verified by MALDI-TOF MS. GLP1_8G37AzF, GLP1_8G37C, and GLP1_8A37C were obtained by processing the respective sfGFP_GLP1 fusion proteins by Factor Xa. The monoisotopic mass of GLP1_8G37AzF, GLP1_8G37C, and GLP1_8A37C were 3474.0, 3414.9, and 3428.7 m/z, respectively (Figure 4). This closely matched the expected values of 3473.6, 3414.7, and 3428.7 m/z, respectively, with less than a 0.1% difference being observed. These results were consistent with the successful construction of the GLP1 variants fused with sfGFP in *E. coli*.

### 3.2. Preparation of Albumin-Conjugated GLP-1 Variants

**Figure 5:**
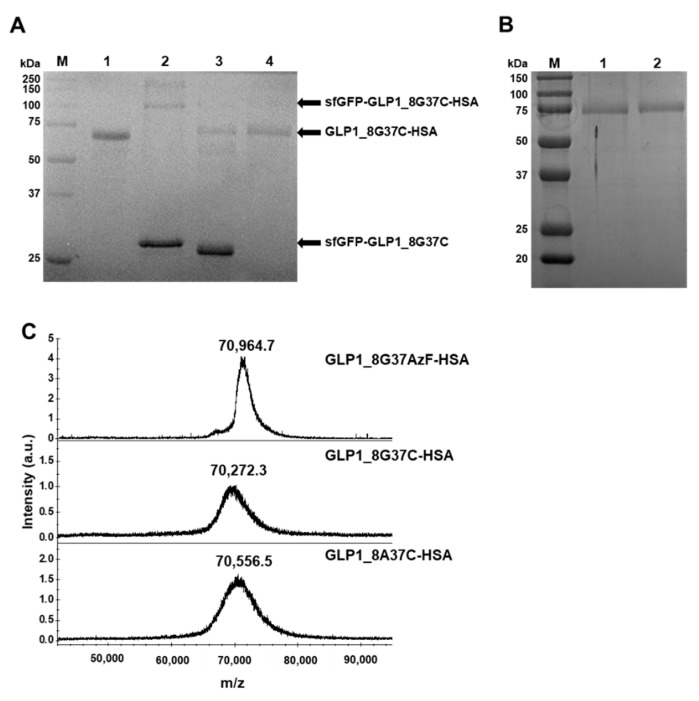
Purification and confirmation of GLP1_8G37C-HSA and their intermediates. (**A**) Protein gel image after Coomassie blue staining of the intermediates during GLP1_8G37C-HSA production. Molecular weight standards (lane M), purified HSA (lane 1), sfGFP-GLP1_8G37C-HSA purified with unreacted sfGFP-GLP1_8G37C (lane 2), GLP1_8G37C-HSA with sfGFP-GLP1_8G37C after factor Xa cleavage (lane 3), and purified GLP1_8G37C-HSA (lane 4). In lane 3, the band for sfGFP-GLP1_8G37C is observed at a lower position compared to the band in lane 2, which is expected to be the result of factor Xa cleavage. (**B**) Protein gel image after Coomassie blue staining of the final products of GLP1_8G37C-HSA (lane 1) and GLP1_8A37CHSA (lane 2). (**C**) MALDI-TOF MS spectrum of GLP1_8G37AzF-HSA, GLP1_8G37C-HSA, and GLP1_8A37C-HSA.

Preparation of the GLP1_8G37AzF-HSA variant was performed as previously reported [24], except for the use of sfGFP-GLP1_8G37AzF instead of sfGFP-GLP1_8G16AzF. Preparation of the HSA-conjugated GLP-1 cysteine variants was initiated by conjugation of a linker to HSA. To confer TCO functionality to HSA, a TCO-MAL linker was used to generate HSA-TCO as we wanted to match the distance between GLP-1 and HSA to the other GLP1-HSA conjugates (GLP1_8G16AzF-HSA and GLP1_8G37AzF-HSA). GLP1_8G16AzF-HSA and GLP1_8G37AzF-HSA have PEG4 and an azide-DBCO complex between GLP-1 and HSA. As TET-PEG4-MAL possesses PEG4, a linker conjugated to HSA does not need PEG. After the generation of HSA-TCO, HSA-TCO was reacted with sfGFP-GLP1_8G37C-TET, resulting in a mixture containing sfGFP-GLP1_8G37C-HSA (Figure 5A). Cation exchange chromatography was performed to isolate sfGFP-GLP1_8G37C-HSA from unreacted albumin (Figure S2). We collected fractions containing sfGFP-GLP1_8G37C-HSA and then proceeded to the next purification step. In the next step, the conjugate was processed by factor Xa, resulting in the GLP1_8G37C-HSA variant, which was purified from the cleaved sfGFP by anion exchange chromatography (Figure S3). The same procedures were used to generate and isolate the GLP1_8A37C-HSA variant. Bands of purified GLP1_8G37C-HSA and GLP1_8A37C-HSA were clearly observed between the 50 and 75 kDa molecular weight standard bands (Figure 5B), which was consistent with the expected molecular weight of ~70 kDa for the GLP1-HSA variants. 

The purified GLP1_8G37AzF-HSA, GLP1_8A37C-HSA, and GLP1_8G37C-HSA were subjected to MALDI-TOF MS analysis (Figure 5C). The observed mass to charge ratios of GLP1_8G37AzF-HSA, GLP1_8G37C-HSA, and GLP1_8A37C-HSA were 70,964.7, 70,272.3, and 70,556.5 m/z, respectively, which were comparable to the expected values of 70,649, 70,782, and 70,796 m/z. The actual ratios compared to expected ratios demonstrated only minor differences (<0.5%). The results of protein gel electrophoresis and MALDI-TOF MS analysis confirmed the successful preparation of GLP1_8G37AzF-HSA, GLP1_8G37C-HSA, and GLP1-8A37C-HSA.

### 3.3. In Vivo Study

**Figure 6:**
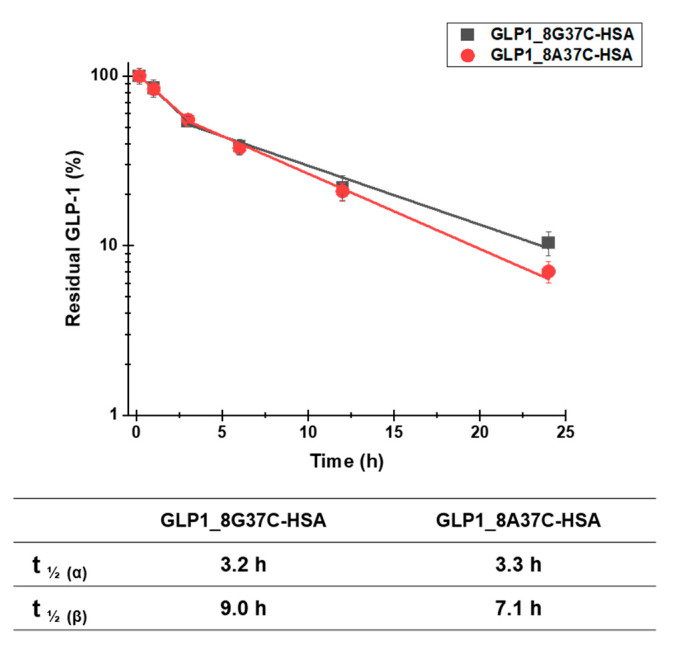
Pharmacokinetic profiles of intravenously injected GLP1_8G37C-HSA and GLP1_8A37C-HSA conjugates in BALB/c mice. Data in the graph indicate the mean ± standard deviation (*n* = 4/group). The plasma concentrations of the samples were plotted using a logarithmic scale.

To investigate the plasma half-lives of GLP1_8G37C-HSA and GLP1_8A37C-HSA, we determined the pharmacokinetic profiles of these variants. Each variant was intravenously administered to BALB/c mice. The serum concentrations of the variants were then evaluated at multiple time points post administration. A sandwich ELISA was used to analyze the serum concentrations. The terminal plasma half-lives of GLP1_8G37C-HSA and GLP1_8A37C-HSA were determined to be 9.0 h and 7.1 h, respectively (Figure 6). The difference is significant (two-tailed student’s *t*-test; *p* < 0.01). The half-lives of these variants were comparable with those of other GLP1-HSA conjugates as previously reported (8.4, 7.4, and 8.0 h for GLP1_8G16AzF-HSA, GLP1_8G19AzF-HSA, and GLP1_8G28AzF-HSA, respectively) [24]. As the site-specific HSA conjugation at positions 16, 19, and 28 of GLP1 led to very similar plasma half-lives [24], we speculated that the terminal plasma half-life of GLP1_8G37AzF-HSA would also be comparable. These results indicated the conjugation of HSA at the C-terminus of GLP-1 successfully extended the plasma half-life of GLP-1. 

Notably, the capture antibody used in the sandwich ELISA to recognize GLP-1 (ABS 033-10-12, Thermo Fischer Scientific) is reported to recognize only the intact N-terminus of GLP-1 (residues 7–17) and does not bind to the DPP-IV-cleaved GLP-1 structure (residues 9‒37). This property of the antibody may explain why the plasma half-life of GLP1_8G37C-HSA was slightly longer than that of GLP1_8A37C-HSA, as GLP1_8A37C-HSA is more vulnerable to DPP-IV cleavage than that of GLP1_8G37C-HSA. 

**Figure 7:**
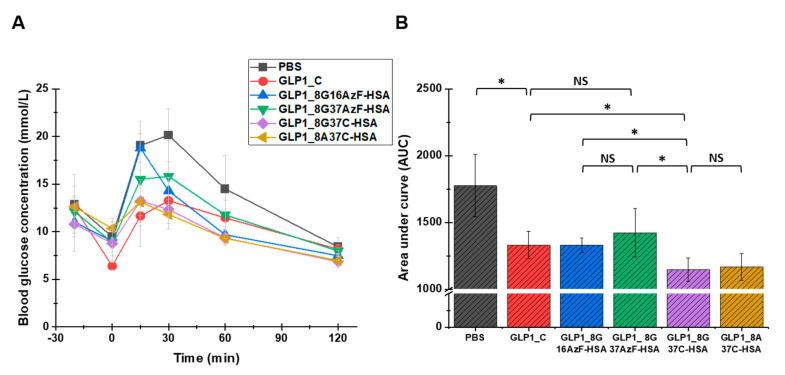
Blood glucose levels of PBS (negative control), GLP1_C (positive control), and the GLP1-HSA variants. (**A**) PBS or 30 nmol/kg doses of GLP1_C or the GLP1-HSA variants were subcutaneously injected into C57BL/6J mice (*n* = 3/group) 20 min prior to the intraperitoneal injection of glucose (1.5 g/kg, 0 min). Data in the graph indicate the mean value ± standard deviation (*n* = 3/group). (**B**) The area under curves (AUC) calculated from 0 to 120 min were compared. Mean value ± standard deviation is presented (*n* = 3/group), * *p*-value < 0.01; NS: not significant.

To evaluate the glucose-lowering activity of the GLP1-HSA variants in vivo, an IPGTT was performed for each of the variants in C57/BL7 mice (Figure 7). In the negative control (PBS) group, blood glucose increased sharply following the glucose injection and slowly returned to normal levels by 120 min after the injection (Figure 7A). As expected, the injection of GLP1_C lowered the blood glucose level. Compared to GLP1_C, GLP1_8G16AzF-HSA was less effective at lowering blood glucose level up to 30 min after glucose injection, but then became more effective after 30 min. Therefore, the in vivo glucose-lowering activities of GLP1_C and GLP1_8G16AzF-HSA were comparable in terms of area under the curve (AUC) values (Figure 7B). Although GLP1_8G37AzF-HSA appeared to be less effective at lowering blood glucose levels than that of GLP1_C and GLP1_8G16AzF-HSA (Figure 7A), its AUC value was not substantially different from those of GLP1_C and GLP1_8G16AzF-HSA (Figure 7B). Therefore, it is clear that a change in the HSA conjugation site from position 16 to position 37 of GLP-1 did not enhance in vivo activity. However, GLP1_8G37C-HSA more effectively lowered blood glucose levels at all times after the glucose injection than GLP1_8G16AzF-HSA and GLP1_8G37AzF-HSA (Figure 7A). Similarly, the AUC value of GLP1_8G37C-HSA was significantly smaller than those of GLP1_C, GLP1_8G16AzF-HSA, and GLP1_8G37AzF-HSA (Figure 7B). GLP1_8A37C-HSA showed very similar patterns regarding blood glucose levels and AUC value compared to that of GLP1_8G37C-HSA. Therefore, both GLP1_8G37C-HSA and GLP1_8A37C-HSA showed the greatest in vivo activities among GLP1_C and the four GLP1-HSA conjugates tested.

As noted in the Introduction, we hypothesized that albumin conjugation to the C-terminus of GLP-1 would reduce the potential steric hindrance of HSA with GLP-1 binding to GLP-1R and enhance in vivo activity, compared to when albumin conjugation is done in the middle of GLP-1. However, when the same bioorthogonal chemistry (SPAAC) used to conjugate albumin to the middle of GLP-1 was used to conjugate it to the C-terminus, in vivo activity was not enhanced. Instead, the change from SPAAC to Michael addition using GLP1_37C variants led to significant enhancement of in vivo blood glucose-lowering activity. This was likely due to reduced steric hindrance caused by the structure obtained after the thiol-maleimide reaction. These results were consistent with previous results that showed even a minor change in the linker or spacer between GLP-1 and the half-life extender can cause considerable differences in biological activity [26].

### 3.4. In Vitro Activity Assay

**Figure 8:**
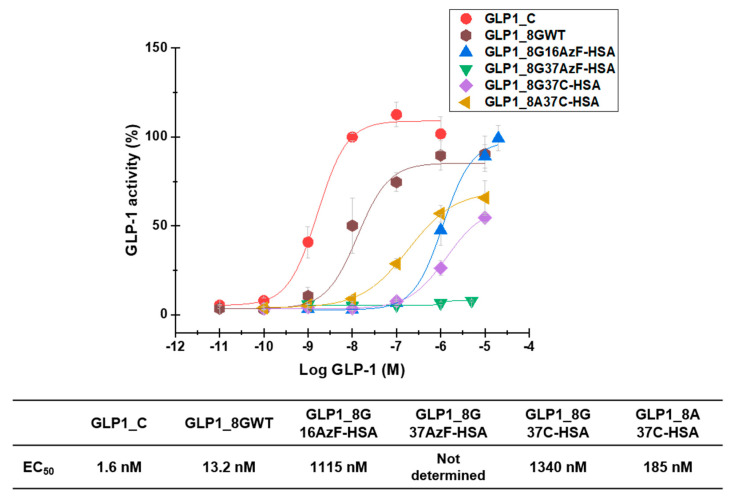
In vitro biological activity of GLP1_C, GLP1_8GWT, GLP1_8G16AzF-HSA, GLP1_8G37AzF-HSA, GLP1_8G37C-HSA, and GLP1_8A37C-HSA evaluated in GLP-1R-expressing HEK-293 cells. The Y-axis denotes the percent activity calculated as a percentage of average cyclic adenosine monophosphate (cAMP) production by 1 μM GLP1_C. Each point in the graph indicates the mean value ± standard deviation (*n* = 3/group).

GLP1-HSA variants were also subjected to in vitro activity assays to evaluate their biological activities. In vitro measurement of cAMP production by GLP-1R-overexpressing HEK-293 cells yielded EC_50_ values of 1.6 nM, 13.2 nM, 1115 nM, 1340 nM, and 185 nM for GLP1_C, GLP1_8GWT, GLP1_8G16AzF-HSA, GLP1_8G37C-HSA, and GLP1_8A37C-HSA, respectively (Figure 8). The EC_50_ value of GLP1_8G37AzF-HSA could not be determined as its activity was too low. 

GLP1_8G37AzF-HSA exhibited very weak in vitro activity (Figure 8), which was consistent with its poor in vivo blood-lowering activity (Figure 7A). The in vitro activity of GLP1_8A37C-HSA was greater than that of GLP1_8G37C-HSA, probably due to the alanine to glycine change at position 8 (A8G) affecting the interactions with GLP-1R. Introduction of the A8G change to GLP1_C also resulted in a similar reduction in in vitro activity, consistent with previous findings [23,25]. The in vitro activity of GLP1-8G37C-HSA was comparable to that of GLP1-8G16AzF-HSA. Despite the low EC_50_ value of GLP1_C, in vivo glucose lowering activity of GLP1_C was inferior to those GLP1-8G37C-HSA or GLP1-8A37C-HSA (Figure 7B) due to its very short plasma half-life (only a few minutes in animal) [32,33]. Some trend differences between the in vitro activities and in vivo activities suggested there are additional mechanisms for in vivo blood glucose level control by GLP-1 beyond the one related to cAMP production. 

## 4. Conclusions

We report the successful preparation of GLP1-HSA conjugates GLP1_8G37C-HSA and GLP1_8A37C-HSA, which exhibited enhanced in vivo glucose-lowering activity by albumin conjugation to the C-terminus of GLP-1 via a thiol-maleimide reaction compared to that of a previously developed GLP1-HSA conjugate (GLP1_8G16AzF-HSA). Another GLP1-HSA conjugate, GLP1_8G37AzF-HSA, prepared by albumin conjugation to the C-terminus of GLP-1 via SPAAC did not exhibit enhanced in vivo glucose-lowering activity. These results indicated that both the albumin conjugation site and conjugation chemistry were important for preparing GLP1-HSA conjugates with enhanced in vivo glucose-lowering activity. Both GLP1_8G37C-HSA and GLP1_8A37C-HSA exhibited prolonged plasma half-lives, comparable to that of GLP1_8G16AzF-HSA, demonstrating the extension of the half-life by albumin conjugation was not significantly affected by albumin conjugate site.

## Data Availability

The supporting data presented in this study are available in the Supplementary Material.

## Supplementary Materials

The following are available online at https://www.mdpi.com/1999-4923/13/2/263/s1, Table S1: Oligonucleotide primers used in this study, Figure S1: Protein gel image of sfGFP-GLP1_8G37C and sfGFP-GLP1_8G37C-TET incubated with or without TCO-Cy5.5, Figure S2: Purification of sfGFP-GLP1_8G37C-HSA after conjugation of sfGFP-GLP1_8G37C-TET to HSA-TCO using IEDDA, Figure S3: Purification of GLP1_8G37C-HSA after factor Xa treatment.
