# Supplementary material for: Effect of C-terminus Conjugation via Different Conjugation Chemistries on In Vivo Activity of Albumin-Conjugated Recombinant GLP-1
Source: Pharmaceutics. 2021 Feb 15;13(2):263. doi: 10.3390/pharmaceutics13020263 (PMC7919490; doi:10.3390/pharmaceutics13020263)
Supplement: Supplementary file 1 [file pharmaceutics-13-00263-s001.pdf]

pharmaceutics

# Supplementary Materials: Effect of C-terminus Conjugation via Different Conjugation Chemistries on In Vivo Activity of Albumin-Conjugated Recombinant GLP-1

Junyong Park, Mijeong Bak, Kiyoon Min, Hyun-Woo Kim, Jeong-Haeng Cho, Giyoong Tae and Inchan Kwon *

**Table S1.** Oligonucleotide primers used in this study.

| Primer Name | Oligonucleotide Sequence (5'→3') | Generated Plasmid |
|---|---|---|
| 16AzF_F | AAGGCACCTTTACCAGCGATTAGAGTAGCTATCTGGAAGG | pQE80-sfGFP-GLP1_16Amb |
| 16AzF_R | CCTTCCAGATAGCTACTCTAATCGCTGGTAAAGGTGCCTT | |
| 37AzF_F | GTGCGGGGGCGTTAGTAAGTCGACCTG | pQE80-sfGFP-GLP1_37Amb |
| 37AzF_R | CAGGTCGACTTACTAACGCCCCCGCAC | |
| G37C_F | GCGGGGGCGTTGCTAAGTCGA | pQE80-sfGFP-GLP1_37Cys |
| G37C_R | ACCAGCCAGGCAATAAATTCTTTGG | |
| A8G_F | GCATCGAAGGTAGGCATGGTGAAGGCACCTTTACCAG | |
| A8G_R | CTGGTAAAGGTGCCTTCACCATGCCTACCTTCGATGC | |

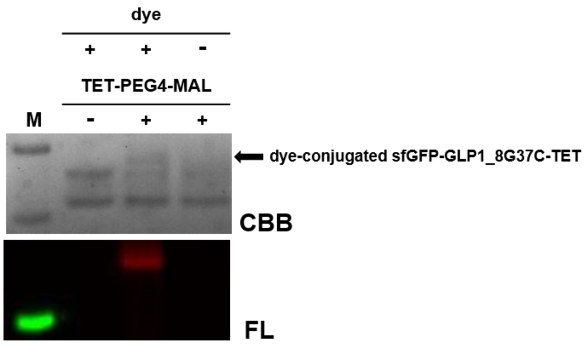


**Figure S1.** Protein gel image of sfGFP-GLP1_8G37C and sfGFP-GLP1_8G37C-TET treated with or without fluorescent dye TCO-Cy5.5. The gel was subjected to irradiation at two ultraviolet wavelengths (302 nm and 620 nm) to excite the fluorophore (FL) and an overlapped image was obtained. The gel was stained with Coomassie blue (CBB) for protein visualization.

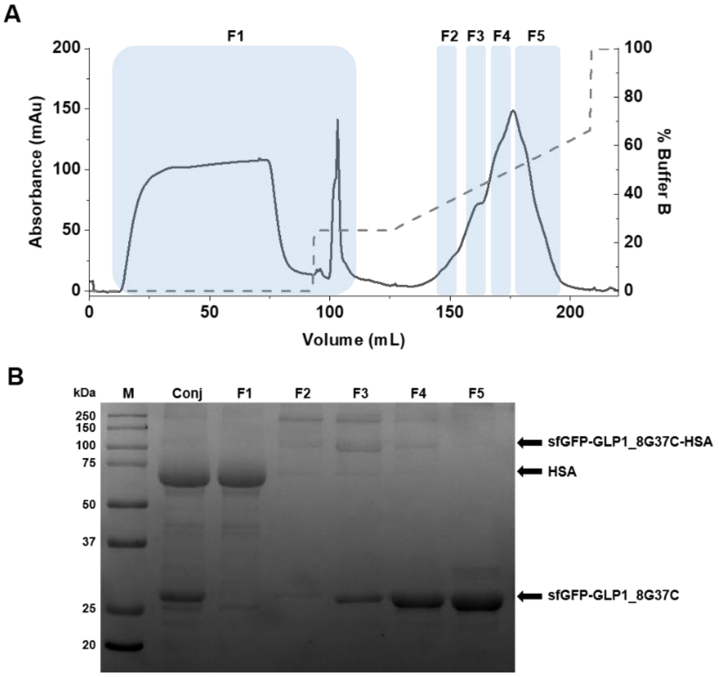


**Figure S2.** Purification of sfGFP-GLP1_8G37C-HSA after conjugation of sfGFP-GLP1_8G37C-TET to HSA-TCO using IEDDA. After conjugation of sfGFP-GLP-1_8G37C-TET to HSA-TCO using IEDDA, cation exchange chromatography was performed to purify the sfGFP-GLP1_8G37C-HSA. (A) Cation exchange chromatogram. The reacted conjugate was desalted using a PD-10 column, loaded onto a HiTrap SP-HP column equilibrated with 20 mM sodium phosphate (pH 6.0), and then eluted with a NaCl gradient. The dotted lines in the chromatogram represents the percentage of Buffer B (20 mM sodium phosphate with 1 M NaCl, pH 6.0). (B) Protein gel image of cation exchange chromatography fractions. The unreacted HSA-TCO unbound to the column is shown in lane F1. There was a prominent peak that seemed to be a mixture of sfGFP-GLP1_8G37C-HSA and unreacted sfGFP-GLP1_8G37C. When analyzed by protein gel electrophoresis, the earlier fractions of the peak (F2, F3) were collected as they showed a more prominent sfGFP_GLP1_8G37C band compared to those of the later fractions (F4, F5). Lane M was loaded with protein molecular weight standards.

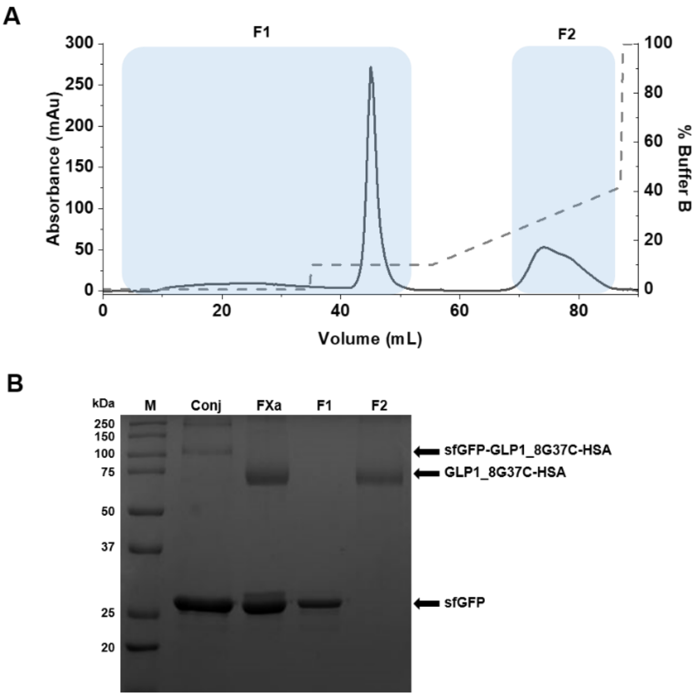


**Figure S3.** Purification of GLP1_8G37C-HSA after proteolytic cleavage by factor Xa. After proteolytic cleavage of sfGFP-GLP1_8G37C-HSA by factor Xa, anion exchange chromatography was performed to purify the GLP1_8G37C-HSA. (A) Anion exchange chromatogram. The previously cleaved mixture of sfGFP and GLP1_8G37C-HSA was desalted using a PD-10 column, loaded onto a HiTrap Q-HP column equilibrated with 20 mM Bis-Tris (pH 6.0), and then eluted with a NaCl gradient. The dotted line in the chromatogram represents the percentage of buffer B (20 mM Bis-Tris with 1 M NaCl, pH 6.0). (B) Protein gel image associated with the anion exchange chromatogram. Protein molecular standards are shown in lane M. Cleaved sfGFP is observed in F1. GLP1_8G37C-HSA is observed in F2, as indicated by the gel image.
